# Costs of delivering human papillomavirus vaccination using a one- or two-dose strategy in Tanzania

**DOI:** 10.1016/j.vaccine.2022.11.032

**Published:** 2023-01-09

**Authors:** Amber Hsiao, Verena Struckmann, Victor Stephani, Devis Mmbando, John Changalucha, Kathy Baisley, Ann Levin, Winthrop Morgan, Raymond Hutubessy, Deborah Watson – Jones, Hilary Whitworth, Wilm Quentin

**Affiliations:** a Department of Health Care Management, Berlin University of Technology, Straße des 17. Juni 135, 10623 Berlin, Germany; b HelloBetter, Oranienburger Str. 86A, 10178 Berlin, Germany; c Mwanza Intervention Trials Unit (MITU), Isamilo Street, P.O. Box 11936, Mwanza, Tanzania; d London School of Hygiene and Tropical Medicine, Keppel St, London WC1E 7HT, London, United Kingdom; e Levin & Morgan, LLC, Bethesda, MD, USA; f Immunization, Vaccines and Biologicals (IVB) Department, World Health Organization (WHO), CH-1211 Geneva 27, Geneva, Switzerland

**Keywords:** HPV vaccination, C4P Tool, Costs, Tanzania

## Abstract

**Objective:**

As part of the Dose Reduction Immunobridging and Safety Study of Two HPV Vaccines in Tanzanian Girls (DoRIS; NCT02834637), the current study is one of the first to evaluate the financial and economic costs of the national rollout of an HPV vaccination program in school-aged girls in sub-Saharan Africa and the potential costs associated with a single dose HPV vaccine program, given recent evidence suggesting that a single dose may be as efficacious as a two-dose regimen.

**Methods:**

The World Health Organization’s (WHO) Cervical Cancer Prevention and Control Costing (C4P) micro-costing tool was used to estimate the total financial and economic costs of the national vaccination program from the perspective of the Tanzanian government. Cost data were collected in 2019 via surveys, workshops, and interviews with local stakeholders for vaccines and injection supplies, microplanning, training, sensitization, service delivery, supervision, and cold chain. The cost per two-dose and one-dose fully immunized girl (FIG) was calculated.

**Results:**

The total financial and economic costs were US$10,117,455 and US$45,683,204, respectively, at a financial cost of $5.17 per two-dose FIG, and an economic cost of $23.34 per FIG. Vaccine and vaccine-related costs comprised the largest proportion of costs, followed by service delivery. In a one-dose scenario, the cost per FIG reduced to $2.51 (financial) and $12.18 (economic), with the largest reductions in vaccine and injection supply costs, and service delivery.

**Conclusions:**

The overall cost of Tanzania’s HPV vaccination program was lower per vaccinee than costs estimated from previous demonstration projects in the region, especially in a single-dose scenario. Given the WHO Strategic Advisory Group of Experts on Immunization’s recent recommendation to update dosing schedules to either one or two doses of the HPV vaccine, these data provide important baseline data for Tanzania and may serve as a guide for improving coverage going forward. The findings may also aid in the prioritization of funding for countries that have not yet added HPV vaccines to their routine immunizations.

## 1 Background

Cervical cancer caused by infection with carcinogenic types of human papillomavirus (HPV) is the fourth leading cancer in women globally [1]. In 2018 about 84 % of all cervical cancers and 88 % of all deaths due to cervical cancer occurred in low-resource countries [1]. In Tanzania, cervical cancer was the most common cancer in women, and there were 9772 new cases and 6695 deaths in the same year [1], [2], [3].

The knowledge that persistent HPV infection is the main cause of cervical cancer has resulted in the development of screening programs and prophylactic vaccines to prevent HPV infection [1]. However, ensuring regular access to and utilization of these services is a particular challenge for health care systems in low- and middle-income countries (LMICs) [4], [5], [6], [7]. Southern and eastern Africa have the highest incidences of cervical cancers because of high rates of HPV infection, inadequate cancer prevention and treatment, low HPV vaccination rates, and limited access to screening programs [1], [3], [8], [9]. Consequently, survival rates are also lower compared to other low-income regions [10].

The World Health Organization (WHO) recommends routine vaccination of all girls aged 9–14 years to protect against HPV infections [11]. Until 2014, the WHO recommended a three-dose HPV schedule, which was then updated to a two-dose regimen when it was demonstrated that antibody responses after two-dose and three-dose schedules were similar after up to five years of follow-up [12], [13]. The change in policy recommendation was meant in part to reduce barriers to immunization, minimize loss to follow-up by requiring one fewer dose, and reduce vaccine cost [12], [14].

Most recently, the WHO’s Strategic Advisory Group of Experts on Immunization (SAGE) concluded based on available evidence that a single-dose regimen of the HPV vaccine provides comparable protection to the two-dose series [15]. A systematic review from Whitworth et al. supports the premise that one dose may be as effective in preventing HPV infection as two or three doses in healthy young females up to seven years post vaccination [13], [16], [17]. Efficacy data from Kenya has also demonstrated that single-dose bivalent and nonavalent HPV vaccines were as effective as multidose regimens [18]. A single-dose regimen would be a more cost-effective policy as vaccine and supply costs typically comprise a substantial portion of a vaccination program’s costs [19], [20], [21]. In resource-limited settings, it would be especially cost beneficial to procure fewer vaccines and thus reduce overall vaccine delivery costs [22], [23].

The Dose Reduction Immunobridging and Safety Study of Two HPV vaccines in Tanzanian Girls (the DoRIS trial; ClinicalTrials.gov NCT02834637) was initiated in 2017 to demonstrate the non-inferiority of immune responses of one dose of HPV vaccine compared with two and three doses of the same vaccine in 930 schoolgirls aged 9–14 years [24], [25], and to immunobridge the immune responses to historical cohorts where efficacy has been demonstrated [26]. Initial results to month 24 after vaccination confirm non-inferiority of a single HPV vaccine dose with either the bivalent vaccine (Cervarix®) or the nonavalent vaccine (Gardasil-9®). These initial results were comparable to those estimated by two observational cohort studies where efficacy with one dose was demonstrated up to 11 years after vaccination [27], [28], [29]. Given that a one-dose strategy would considerably reduce the costs of vaccination, the study has important implications for cost-effectiveness of the vaccine, which were assessed in the economic component of the trial. As part of the economics component of the DoRIS trial, the current costing study aimed to estimate the costs of alternative dosing strategies of a national HPV vaccination program in Tanzania from the perspective of the Tanzanian government. Alongside the DoRIS trial, Tanzania also implemented its national HPV vaccination program in 2018 using a two-dose quadrivalent HPV vaccine given to girls aged 14 years, which provided the opportunity to study the costs of one of the first national HPV vaccination programs in Africa.

Previous studies have reported cost data regarding HPV vaccination demonstration projects [20], [30]. The current study is one of the first to estimate the costs of an existing national HPV vaccination program. We aimed to: (1) estimate financial and economic costs of a two-dose vaccination program based on experiences with the national vaccination program that took place in schools, health facilities, and mobile events, (2) estimate costs of a one-dose vaccination schedule to enable future cost-effectiveness analyses, and (3) assess the effect of alternative assumptions for future vaccination coverage rates on estimated costs of vaccination.

## 2 Materials and methods

The WHO Cervical Cancer Prevention and Control Costing (C4P) micro-costing tool [31]—developed to assist low middle-income countries in cervical cancer prevention planning—was used to estimate the incremental financial and economic costs of the national vaccination program from the perspective of the Tanzanian government. The HPV vaccination module of the tool enables users to project future costs or retrospectively evaluate prior costs of an HPV program. The tool outputs the total cost, cost per fully immunized girl (FIG), and cost per vaccine dose.

The costs are further broken down by financial and economic costs. Incremental financial costs represent actual expenditures on goods and services, while incremental economic costs represent the economic value of all resources used including costs of goods and services that do not require additional financial resources (e.g., salaries of existing staff). **Supplemental** Table 1 provides required details on all inputs used in the tool for this analysis.

### 2.1 Data collection

Data were collected in 2019 from four different sources (Table 1): (1) national cost and coverage reports (including Ministry of Health, Community Development, Gender, Elderly, and Children [MoHCDEC] data), (2) three costing workshops with stakeholders in Dar es Salaam, (3) interviews with national, regional, and district stakeholders, as well as health facility personnel in the Mwanza region, and (4) observation of health workers in selected health facilities in the Mwanza region.

**Table 1:** Overview of data sources.

Name or Source	Explanation/ Definition	Data collected through source (examples)
National/ international reports	Budget report, Ministry of Health, Community Development, Gender, Elderly, and Children [MoHCDEC], Ministry of Education (BEST report), *EPI* – DMT report, National Bureau of Statistics, Bank of Tanzania, World Bank, World Health Organization	Number of schools, number of schoolgirls, number of girls aged 14 years, annual population growth rate, proportion of in-school girls, salary scales, microplanning, sensitization, number of districts, number of health facilities in Tanzania, annual discount rate, annual exchange rate, HPV coverage, and drop-out rates
Costing workshop	Different stakeholder groups (e.g. MoHCDEC, Immunization and Vaccines Development (IVD) program, WHO, US Centers for Disease Control and Prevention (CDC), international development partners) met during three workshops to estimate future HPV vaccination costs using the C4P tool	Vaccines and supplies, proportion of vaccinations delivered by a selected strategy, national supervision, supervision costs, allowances (supervision), cold chain, training, proportion of HPV-related training workshop time
Interviews with national/regional stakeholders and health facility personnel	Interviews were conducted by research staff during field visits	Mwanza Intervention Trials Unit car costs, training, supervision costs, school delivery approaches, regional microplanning report, regional training costs, vaccine distribution costs
Ring fencer questionnaire/ observations	Trained staff employed by the DoRIS study to ensure that girls who were included in the trial did not receive an additional dose through the national program. Ring fencers observed the work of regular nurses in health facilities and schools	Salary categories, travel times to schools, vaccination times at schools and health facilities per vaccinee, average number of vaccinations delivered at schools, supervision visits, teachers’ and nurses’ allowances, average number of vaccinators per school visit

National and international cost and coverage reports were systematically reviewed for data extraction (using pre-prepared data forms) on the number of girls aged 9–14 years enrolled at school or dropped out. HPV program data were also extracted from these sources, including the number of girls vaccinated with one or two doses of HPV vaccine in 2018 and 2019.

Data collection coincided with three cervical cancer costing workshops organized by the WHO in Dar es Salaam for various stakeholders (e.g., MoHCDEC, WHO) in February, April, and November 2019. The aim of these workshops was to estimate future costs of cervical cancer prevention and control. Information was collected regarding costs of vaccines and injection supplies, microplanning, training, sensitization, service deliver, supervision, and cold chain. Data retrieved from the costing workshops were used as the basis for completing the C4P tool (see Table 1).

Interviews with experts (“key informants”) in this study were conducted to gather information on various aspects of the existing national vaccination program, including service delivery, coverage, training, sensitization, and cold chain logistics, management, and supervision. All interviewees were comfortable being interviewed and responding in English. These key informant interviews targeted various administrative levels: district immunization and vaccine officers (DIVO), regional immunization and vaccine officers (RIVO), and local health professionals (vaccination nurses). Interviews were carried out face-to-face when feasible; otherwise, interviews were conducted via phone/e-mail/Skype. This allowed for a better understanding of the overall vaccination process already in place and formed the basis for adjusting the C4P tool in line with experiences from the national HPV vaccination program.

Finally, DoRIS trial staff members (“ring-fencers” who were primarily employed to ensure that girls who were vaccinated in the DoRIS study did not also receive additional HPV doses through the national vaccination program) collected more specific data on vaccination processes in the Mwanza region. They observed vaccinations at nine health facilities and 10 schools to obtain more accurate estimates of service delivery times. In addition, they collected information on salary categories of vaccinating nurses, allowances for teachers and nurses, and average numbers of vaccinators who visited schools.

### 2.2 Data analysis

The costs were collected in Tanzanian shillings (TSh) and converted to US dollars (US$), using the official exchange rate by the World Bank for the year 2019 ($1USD = 2288.208TSh). Costs were categorized as one of the following vaccination activities: vaccine and injection supplies, cold chain expansion, microplanning, training, sensitization, social mobilization, service delivery, and supervision. Financial and economic costs of all activities were calculated and compared.

Financial costs only included new expenditures paid for by the MoH to implement the program, such as injection supplies, training resources, and outreach allowances. Economic costs included all financial costs, as well as the value of other resources used for vaccine introduction that may have already been paid for or owned by the MoH, such as existing cold chain used for other vaccines, health personnel salaries, and volunteer time. As an example, the cost of donated vaccine would be $0 in the financial costing since it was not paid for by the MoH, but would be fully accounted for at $4.50 per dose in the economic costing. If vaccines were procured by the MoH, then vaccine costs would be accounted for in both the financial and economic costing.

Within the financial and economic categories, costs were further differentiated as introduction and recurrent costs (operational). Introduction costs included the value of resources incurred at HPV vaccination program introduction, such as initial trainings and capital goods (that last longer than 1 year, e.g., cold chain equipment and vehicles). Recurrent costs included the value of resources that last less than a year, such as personnel time, vaccines, and transportation. These recurrent costs are captured in the C4P tool as vaccine and injection supplies, training, service delivery, supervision, and cold chain.

Straight-line depreciation was used for financial costs (cost divided by useful lifespan in years of good), which assumes capital goods (e.g., cold chain equipment) are used equally over time. Economic costs were calculated using a standard 3 % discount rate, as well as a sensitivity analysis scenario that tested alternative assumptions (see next section) [21], [32].

The basic assumptions of incurred costs of the national HPV vaccination program are shown in **Supplemental** Table 1. We assume in our base case scenario that each year, 40 % of vaccines are delivered at health facilities, 55 % at school-based events, and 5 % at mobile events. For this analysis, coverage was defined as single-dose coverage and second-dose coverage; dropout was defined as the difference between the first and second dose. The cost per fully immunized girl (FIG) was calculated by dividing total 5-year program costs by the number of estimated FIG over the 5-year period; costs per dose were calculated by dividing the number of estimated doses delivered over the 5-year period.

Based on costing workshop assumptions, 60 % of the introduction costs (e.g., health care provider training, microplanning, sensitization, and social mobilization) were allocated to HPV vaccination and 40 % to the inactivated polio vaccine (IPV) vaccination program that was introduced in Tanzania in the same year that the DoRIS trial began.

### 2.3 Sensitivity analysis

Deterministic sensitivity analyses (SA) were performed to assess the effect of alternative assumptions for vaccination coverage, the parameters within each delivery strategy (e.g., vaccination time and number of vaccinees within school-based events), and the impact of a potential one-dose vaccination schedule were also varied. **Supplemental** Table 2 details each parameter that was varied.

Univariate and multivariate SA were carried out using best-case and worst-case scenario assumptions. For the univariate sensitivity analysis, one model parameter was varied at a time while other parameters remained constant. For the multivariate SA, all variables were simultaneously set to the best-case assumptions or worst-case assumptions, respectively, in order to generate the largest possible range of costs in an analysis of extremes [21], [33].

Base case assumptions for HPV vaccination coverage and dropout rates were based on actual coverage numbers from 2018 and 2019 provided by Tanzania’s *EPI* [34], and for subsequent years, data from the costing workshop (**Supplemental** Table 2). Best-case assumptions were based on other vaccine coverage rates in Tanzania and on HPV vaccination coverage rates from other African countries (85 % in 2020, 90 % in 2021, and 95 % in 2022) [35]. The worst-case assumptions (78 %) were based on WHO coverage data for 2018 and 2019 and assumed no coverage increase in subsequent years. The base case scenario regarding the proportion of vaccines delivered via each delivery strategy remained unchanged in sensitivity analyses.

Base case service delivery cost assumptions were based on duration of time spent on HPV vaccination observed by ring fencers in health facilities. Best-case assumptions were based on costing workshops and worst-case assumptions reflected individual vaccination sessions observed by ring fencers. In the base case scenario, the round trip travel time to and from school and time spent administering vaccinations in schools were also based on ring fencer observations and included an assumption regarding the average number of persons vaccinated, with one or two vaccinators in attendance (1.62 on average), which was typical of what was observed in the field. Best-case assumptions used the same length of time for school vaccinations (157 mins), but assumed only one vaccinator in attendance (see Supplemental Table 2). Worst-case assumptions were based on the costing workshop and assumed that vaccinators worked a full day (8 h) at the school vaccination event or did not resume work at the health facility after the event.

## 3 Results

### 3.1 Total costs

The total financial and economic costs were US$10,117,455 and US$45,683,204, respectively (Table 2) in the base case scenario where vaccines are distributed in health facilities, schools, and mobile events, with nearly 4.57 million girls vaccinated (Table 3). Vaccine and injection supplies were the main contributors to total costs, accounting for 46 % of financial costs and 55 % of economic costs. The total estimated cost for procuring 4.6 million doses of HPV vaccine (and injection supplies) in Tanzania between 2018 and 2022 was estimated at US$4.6 million (financial) and US$25.3 million (economic). Apart from vaccine and injection supply costs, service delivery was the main contributor to financial costs at 38 %, while cold chain and service delivery costs were the main contributors to economic costs at 23 % and 16 %, respectively. Introduction costs, i.e., microplanning, training, and sensitization, comprised about 9 % of total financial costs (US$922,575) and 3 % of economic costs (US$1,586,222).

**Table 2:** Estimated financial and economic costs of the national immunization [33]–2022 US$2019.

	**Costs (US$2019) by category and % of total**					
	**2018**	**2019**	**2020**	**2021**	**2022**	2018–2022*
**Financial Costs (Total)**	**$2,200,947**	**$1,665,788**	**$1,861,940**	**$2,084,785**	**$2,303,994**	**$10,117,455**
Vaccine and Injection Supplies	$619,399 (28 %)	$828,190 (50 %)	$935,293 (50 %)	$1,057,337 (51 %)	$1,176,609 (51 %)	**$4,616,828 (46 %)**
Microplanning	$125,411 (6 %)	—	—	—	—	**$125,411 (1 %)**
Training	$405,412 (18 %)	—	—	—	$1,708 (<1%)	**$407,121 (4 %)**
Sensitization	$390,043 (18 %)	—	—	—	—	**$390,043 (4 %)**
Service Delivery	$523,131 (24 %)	$700,047 (42 %)	$789,097 (42 %)	$889,898 (43 %)	$988,126 (43 %)	**$3,890,300 (38 %)**
Supervision	$89,669 (4 %)	$89,669 (5 %)	$89,669 (5 %)	$89,669 (4 %)	$89,669 (4 %)	**$448,343 (4 %)**
Cold Chain	$47,882 (2 %)	$47,882 (3 %)	$47,882 (3 %)	$47,882 (2 %)	$47,882 (2 %)	**$239,408 (2 %)**
**Economic Costs (Total)**	**$8,254,230**	**$8,110,586**	**$8,915,448**	**$9,778,181**	**$10,624,759**	**$45,683,204**
Vaccine and Injection Supplies	$3,394,445 (41 %)	$4,538,668 (56 %)	$5,125,614 (58 %)	$5,794,442 (59 %)	$6,448,083 (61 %)	**$25,301,253 (55 %)**
Microplanning	$150,494 (2 %)	**—**	**—**	**—**	**—**	**$150,494 (<1%)**
Training	$866,148 (11 %)	**—**	**—**	**—**	$4,018 (<1%)	**$870,166 (2 %)**
Sensitization	$565,563 (7 %)	**—**	**—**	**—**	**—**	**$565,563 (1 %)**
Service Delivery	$1,007,757 (12 %)	$1,348,639 (17 %)	$1,520,012 (17 %)	$1,713,917 (18 %)	$1,902,836 (18 %)	**$7,493,161 (16 %)**
Supervision	$136,201 (2 %)	$136,201 (2 %)	$136,201 (2 %)	$136,201 (1 %)	$136,201 (1 %)	**$681,003 (1 %)**
Cold Chain	$2,133,622 (26 %)	$2,087,078 (26 %)	$2,133,622 (24 %)	$2,133,622 (22 %)	$2,133,622 (20 %)	**$10,621,565 (23 %)**

*Totals may not sum to 100% due to rounding.

**Table 3:** Number of vaccinated girls in school and out-of-school (health facilities and mobile events) by dose, 2018–2022.

	**2018**	**2019**	**2020**	**2021**	**2022**	2018–2022*
**In school**						
HPV first dose	228,790	311,543	337,345	360,177	379,712	**1,617,567**
HPV second dose	151,002	196,272	236,141	288,142	341,740	**1,213,297**
**Out-of-school**						
HPV first dose	140,226	190,946	206,760	220,754	232,727	**991,412**
HPV second dose	92,549	120,296	144,732	176,603	209,454	**743,634**
**Total**						
**HPV first dose**	**369,017**	**502,488**	**544,104**	**580,931**	**612,438**	**2,608,979**
**HPV second dose**	**243,551**	**316,568**	**380,873**	**464,745**	**551,194**	**1,956,931**

*Totals may not sum to 100% due to rounding.

## 4 Costs per fully-immunized girl (2 doses)

**Fig. 1:**
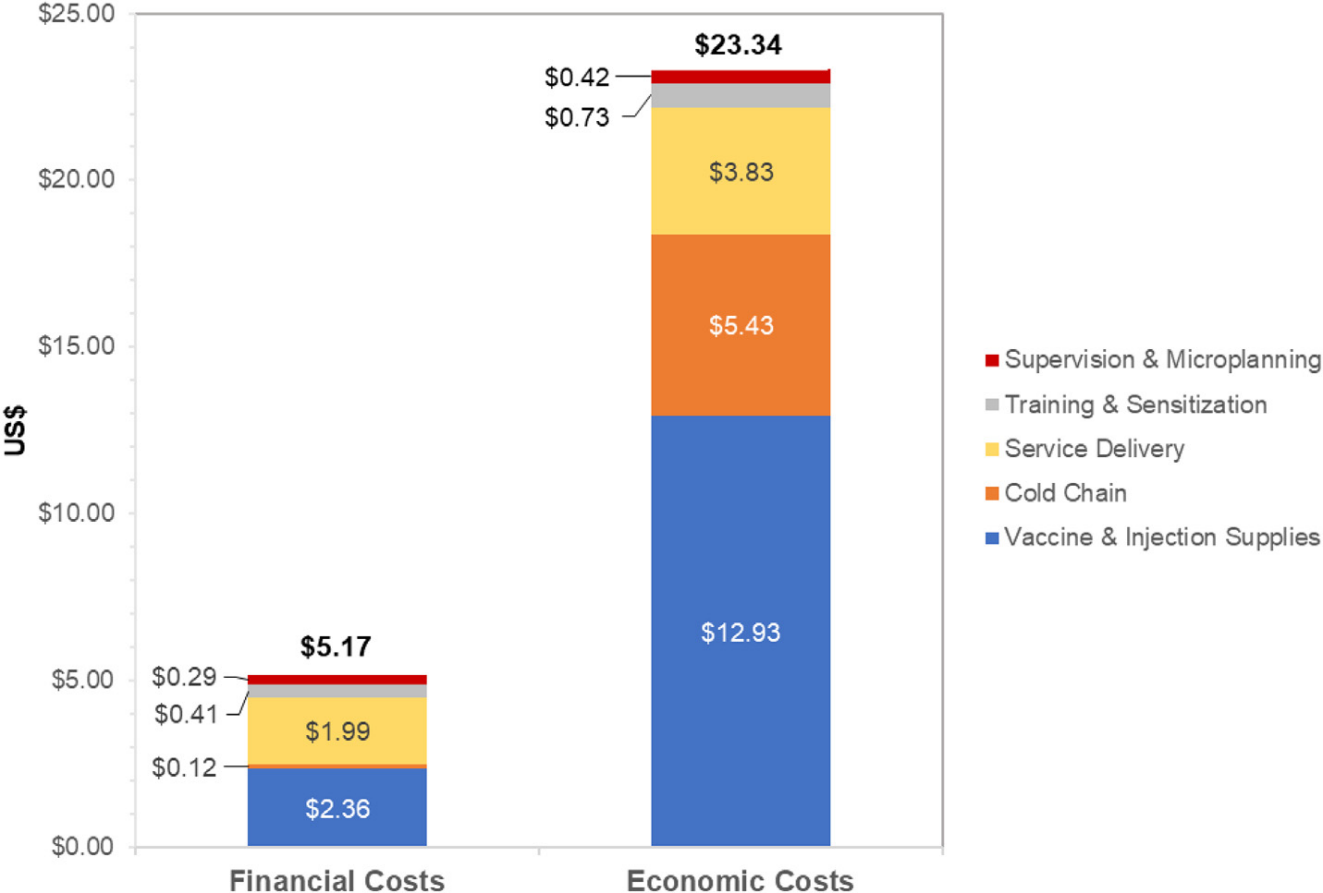
Estimated financial and economic costs in US$2019 per fully immunized girl (two-doses).

Costs per dose were US$2.22 (financial) and US$10.01 (economic). Assuming a 2-dose fully immunized scenario, costs per FIG were US$5.17 (financial) and US$23.34 (economic) (Fig. 1).

### 4.1 Costs per fully-immunized girl (1 dose)

**Fig. 2:**
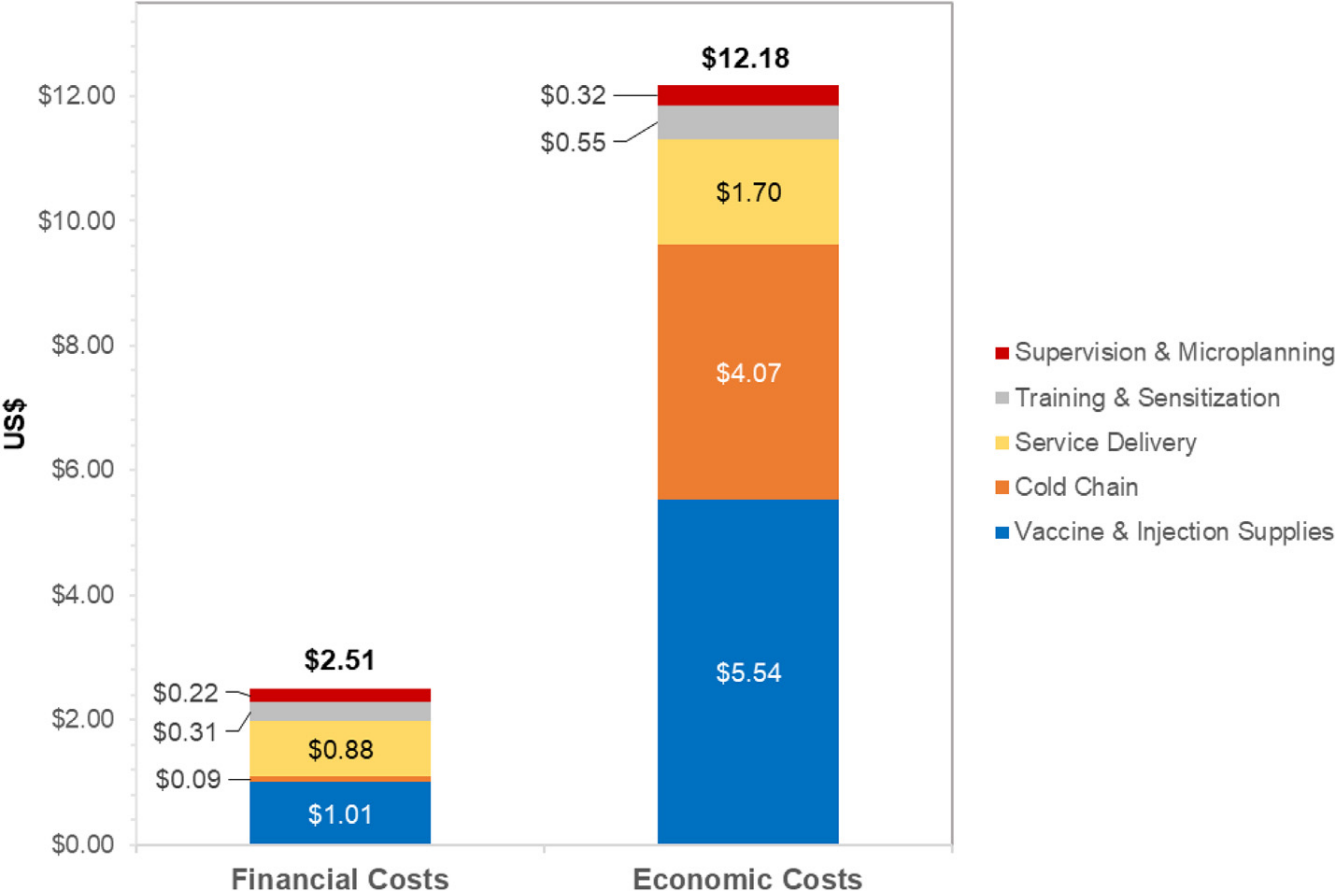
Estimated financial and economic costs in US$2019 per fully immunized girl (hypothetical one-dose scenario).

If one dose of HPV vaccine were sufficiently protective, costs per dose (and costs per FIG) would be $2.51 in financial costs (compared to $5.17 for two doses) and $12.18 in economic costs (compared to $23.34 for two doses) (Fig. 2). This would be a reduction of 51 % in financial and 48 % in economic costs compared to the two dose per FIG scenario. For economic costs, a one-dose scenario results in large reductions in costs to vaccine and injection supplies (-57 %), service delivery (-56 %), and cold chain (-25 %), when compared with the two-dose scenario.

### 4.2 Sensitivity analysis

**Fig. 3:**
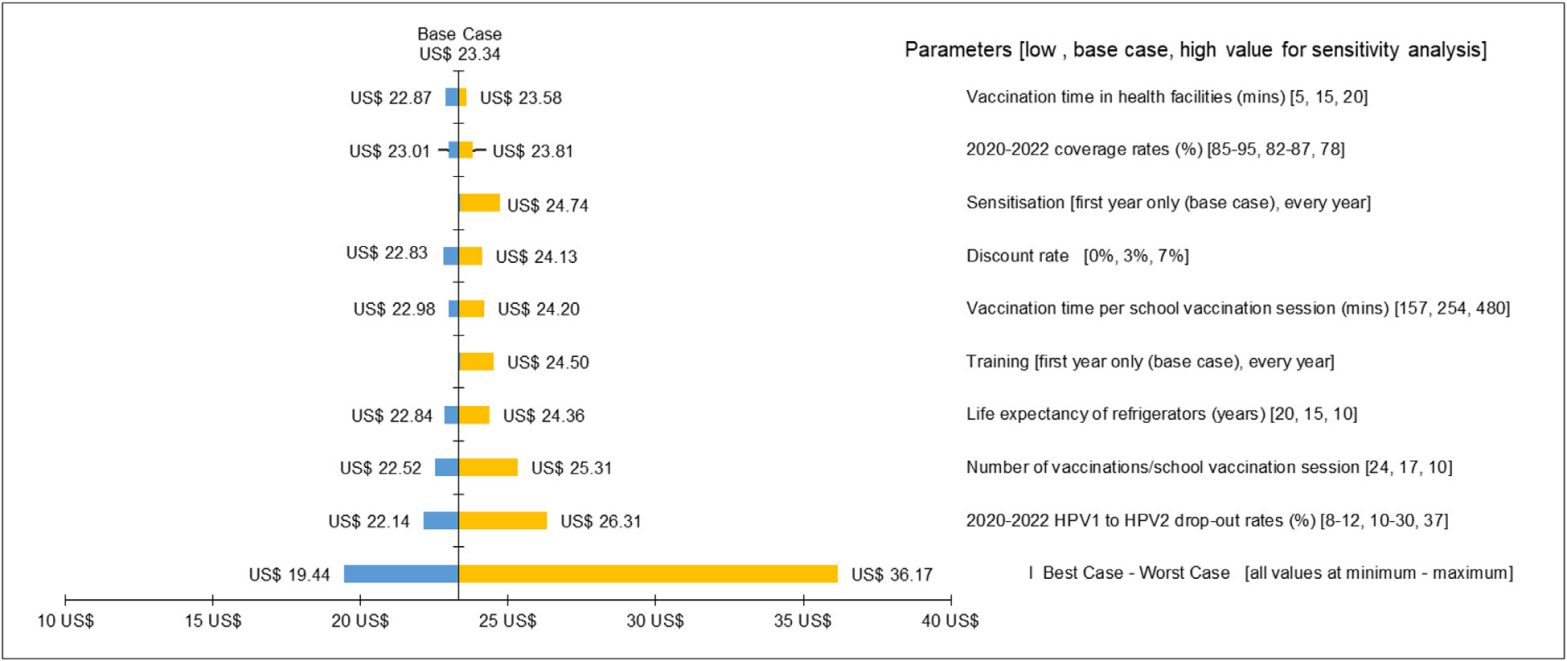
Sensitivity analysis of economic costs in US$2019 per fully immunized girl (two-doses).

The economic costs per fully immunized girl (two doses) ranged from $19.44 in a best-case scenario to $36.17 in a worst-case scenario (Fig. 3). In univariate analyses, costs were most sensitive to varying the first to second dose drop-out rate between 2020 and 2022. If drop-out rates remained at 37 % in all years (as observed in 2019), costs per girl would increase to $26.54, while costs would reduce to $22.14 if drop-out rates were reduced to 8 % in 2022 (as assumed by experts at the costing workshop). The number of vaccinees per school vaccination session also had a considerable impact on estimated costs, with estimates ranging from $22.52 if 24 girls were vaccinated per school session to $25.31 if only 10 girls were vaccinated per session. Costs were relatively insensitive to varying the discount rate (range: $22.83–$24.13).

In multivariate analysis when all variables were simultaneously set to the worst-case assumptions, the costs were nearly 55 % higher ($36.17) than the base case scenario ($23.34).

## 5 Discussion

Using a standardized HPV costing tool, this study was the first to analyze the cost of a national HPV vaccination program in school-aged girls in Africa. This study found that the overall cost of Tanzania’s HPV vaccination program was lower per vaccinee than previous demonstration projects in the region suggest [20], [30], at a financial cost of $5.17 per two-dose fully vaccinated girl (FIG), and $2.51 per one-dose FIG. When economic costs are considered (which include financial costs), the cost per vaccinee increased to $23.34 per two-dose FIG and $12.18 per one-dose FIG.

Vaccines and vaccine-related costs, such as syringes and waste containers, were the main driver of financial and economic costs at nearly 46 % and 55 % of costs, respectively. Our sensitivity analysis found that the drop-out rate from first to second dose and the efficiency of service delivery had the largest impact on costs—the more girls vaccinated per session, the lower the cost per fully vaccinated girl. Thus, the efficiency of school-based delivery methods was heavily dependent upon the number of girls vaccinated during a session. In multivariate sensitivity analysis, overall costs increased by more than $10 when all model parameters were set to the highest cost assumptions, which would be a significant increase.

As expected, service delivery was also a substantial portion of financial costs (38 %), given the upfront investments necessary to scale the HPV vaccination program nationally to an age group that was previously not routinely targeted for vaccination. Further, given the age cohort targeted, additional resources had to be allocated to reach both primary and secondary schools (given that girls aged 14 years may be in either setting) [36].

Our findings are similar to a recent study estimating costs of cervical cancer prevention and control in Tanzania for the period 2020–2024 [30]. The study used HPV vaccination costs of $6.68 in financial costs (vs $5.17 in DoRIS) and $17.31 in economic costs (vs $23.34 in DoRIS), with some notable differences that explain the larger discrepancy in economic costs especially. The current study assumed lower vaccination coverage based on national data (59–87 %), whereas Levin et al. estimated future vaccine coverage based on in-country expert opinions (80–90 %). Vaccine costs were the same ($4.50), but the current study assumed a subsidy of $4.30, which was the Gavi, the Vaccine Alliance, subsidy received by Tanzania at the time. Levin et al. used a subsidy of $3.60 (80 % of costs), which would be the Gavi subsidy for countries in “preparatory transition” to self-funding starting at a GNI above $995. Further, given existing cold chain in the country for other vaccines, Levin et al. did not account for these costs whereas for the DoRIS study, we assumed that HPV vaccine-related activities, such as vaccine delivery and storage, used a portion of the existing cold chain (i.e., economic costs of these resources). There were also differences in assumptions regarding vaccinator time and salaries, based on what was reported by workshop attendees for each respective study. Finally, while Levin et al. only included national supervision costs, the current study also estimated regional- and district-level supervision costs.

Quantification of the costs has important implications for Tanzania, as well as other countries that may want to introduce HPV into their routine immunization schedules. Since its introduction of its pilot in the Kilimanjaro region followed by the national HPV vaccination expansion, both first and second dose coverage rates have decreased over time [37]. While there is limited evidence of comparable efficacy in immunocompromised populations, the recent SAGE recommendation to use either a one- or two-dose schedule for most individuals will allow countries to have greater flexibility to more rapidly scale-up HPV vaccination programs and increase overall vaccination coverage [15]. At roughly half the cost (financial: $2.51; economic: $12.18), this would result in significant cost savings, or an increase in the number of girls that could be reached (assuming the same amount of vaccine supply as a two-dose series). It would also reduce the complexities associated with lost to follow-up and appropriate timing for second dose administration.

The economic cost per dose for HPV vaccination of $12.18 in this study is higher than what has been previously estimated as the cost per dose for various routine vaccinations in Tanzania. One study estimated the economic cost of the measles-rubella vaccine as $4.35 per dose in US$2019 (reported as $3.99 in US$2016) [37]. Another study estimated an average economic cost of $4.71 per dose in US$2019 (reported as $4.32 in US$2016) for all routine childhood vaccines [38]. This difference is likely due at least in part to the target age group and start-up costs associated with reaching a new age group. When compared to the economic cost of cervical cancer treatment, which is nearly $100 for pathology and several hundreds of dollars more for chemotherapy and radiotherapy [39], the cost of immunizing girls against HPV is a worthwhile investment.

While our findings are promising, a successful long-term vaccination program requires significant financial commitment and more targeted outreach beyond its initial introduction. A recent feasibility assessment found that while the HPV vaccine and its safety is well accepted among Tanzanians, there are gaps in knowledge and misinformation in the community that could be detrimental to the country’s progress [37]. Studies in other African countries have also stressed the importance of increasing knowledge and awareness of HPV and cervical cancer [40], [41], [42]. Further, community leaders were found to have low basic knowledge of HPV vaccination, which points to the need for additional investments in community engagement and outreach that are not included in our modelling. The reality is that at age 14, 36 % of Tanzanian girls are already out-of-school, and 45 % at age 15 (compared with only 4 % of girls aged 9 years in the Kilimanjaro demonstration project) [36]. The poorest children and those living in rural areas face additional disparities in primary school attendance. The cost-saving one-dose strategy could help alleviate such disparities by making more vaccine doses available and increasing vaccine delivery capacity to better serve hard-to-reach groups of girls [43].

This study had limitations. Though efforts were made to use the best available data, some of the model inputs were more robust than others. For example, some costs collected via the costing workshop may have been subject to recall bias (e.g., estimates regarding frequency and length of HPV-specific trainings), or may be more highly variable depending on region (e.g., travel allowances and travel time). On the other hand, vaccine and supply costs make up a substantial portion of program costs and are likely more stable. Additionally, in its current form, the HPV vaccination program only targets the age 14 cohort given limited vaccine supply; once more vaccine is available, more resources—service delivery in particular—will be needed to reach more girls, though the program may be more cost-effective at that point, given that vaccinators can focus on younger cohorts (e.g., age 9) who are more likely to be in school. Focusing on earlier ages in the recommended range for vaccination may also reduce the need to target secondary schools.

As part of its global strategy to eliminate cervical cancer, the WHO has set the goal of having 90 % of girls in all countries be fully vaccinated by the time they reach age 15 years by 2030 [44]. This is a highly ambitious goal, given the limited supply of HPV vaccines globally and disparities in access, especially in developing countries. As of 2020, < 25 % of low-income and < 30 % of lower-middle-income countries had introduced the vaccine into their vaccination programs [44]. Further, a recent *meta*-analysis of HPV vaccination uptake in low- and middle-income countries found that in many of these countries, coverage was high initially due to the initial investments in demonstration projects by international non-governmental organizations, such as the Gardasil Access Program [45], the Program for Appropriate Technology in Health (PATH), and Gavi. Once these projects withdrew, uptake dropped substantially [45]. In order for countries to reach the WHO goal, it is necessary for them to prioritize spending on HPV (and other) vaccination programs to ensure their success.

## 6 Conclusions

In summary, this study evaluated the financial and economic costs of the national rollout of the HPV vaccination program in Tanzania. The overall cost of Tanzania’s HPV vaccination program was lower than previously estimated, and a single-dose scenario has the potential to increase vaccination coverage substantially without increasing cost. These data provide a baseline to aid in the prioritization of spending for the program in the coming years and may also serve as a guide for other countries that have yet to introduce HPV vaccines.

## Funding

The DoRIS trial is funded by the Joint Global Health Trials Scheme (MR/N006135/1) of the UK Department of Health and Social Care (DHSC), the Foreign, Commonwealth & Development Office (FCDO), the Global Challenges Research Fund (GCRF), the Medical Research Council (MRC), and Wellcome as well as by the Bill and Melinda Gates Foundation (OPP1167526). The funders did not have a role in the design of the study, collection, analysis, interpretation of the data, and writing of the manuscript.

## Authors’ contributions

WQ, RH, and DW conceptualized the study. WQ, V Stephani, DM, KB, and HW performed data collection. V Struckmann, V Stephani, WM, and WQ performed the data analysis. AH, WQ, and DW reviewed and interpreted the data. AH, WQ, and VS drafted the manuscript. AH, WQ, AL, DW substantively revised and finalized the manuscript. All authors approved the final version for submission.

## Declaration of Competing Interest

The authors declare that they have no known competing financial interests or personal relationships that could have appeared to influence the work reported in this paper.

## Data Availability

Data will be made available on request.
