# Supplementary material for: Costs of delivering human papillomavirus vaccination using a one- or two-dose strategy in Tanzania
Source: Vaccine. 2023 Jan 9;41(2):372–9. doi: 10.1016/j.vaccine.2022.11.032 (PMC9831118; doi:10.1016/j.vaccine.2022.11.032)
Supplement: Supplementary data 1 [file mmc1.docx]

**Supplemental Table 1.** C4P tool inputs for main analysis

| **Category** | **Input** | **Multiplier** | **Additional multiplier (if applicable)** | **Data source and notes** |
| --- | --- | --- | --- | --- |
| **Population Data** |  |  |  |  |
| National HPV vaccination coverage, 2018 | 59% | — | — | Tanzanian EPI Programme; HPV 1 & 2 Vaccination Performance, December 2018 |
| Second dose coverage | 25% | — | — | Tanzanian EPI Data Management Tool (DMT); Coverage in September-December 2018 |
| National HPV vaccination coverage, 2019 | 85% | — | — | Tanzanian EPI Programme; HPV 1 & 2 Vaccination Performance, December 2019 |
| Dropout rate (First to second dose) | 51% | — | — | Tanzanian EPI Data Management Tool (DMT); Dropout rate of 51% based on coverage difference from March-June 2018 compared with September-December 2018 |
| Number of 14-year-old girls | 625,452 | girls | 3% | Tanzanian EPI report 2018; assumed 3% annual population growth rate |
| Proportion of 14-year-old girls in school | 62% | — | — | Ministry of Education (BEST report 2016) reports 38% out of school |
| **Vaccines** |  |  |  |  |
| Vaccine and syringes | $4.30 (subsidized) $0.20 (financial) | — | — | Costing workshop; includes vaccine and injection syringe per dose (subsidized price paid by international donors; financial cost of $0.20) |
| Safety box for syringe disposal | $0.80 (subsidized) $0.00 (financial) | — | — | Costing workshop; assumes 100 syringes may be disposed per box (subsidized price of $0.80 paid by international donor $0.80; thus financial cost of $0) |
| Freight, handling, insurance costs for vaccine shipment | 4%-7% | — | — | Costing workshop; additional costs for this category were a proportion of vaccine costs (not including syringe and safety boxes) |
| Projected proportion of vaccines, syringes, and safety boxes wasted | 3% | — | — | Costing workshop |
| Cotton pads (500 gm roll) | $1.40 | 1% for HPV | — | Costing workshop and UNICEF supply catalog |
| **Cold chain** |  |  |  |  |
| Refrigerators | 485.008487 | 22% | — | Costing workshop; 22% of existing refrigerator space allocated to HPV vaccines |
| Incinerators (annualized cost) | 366.0793949 | — | — | Costing workshop; an estimated two-thirds of health facilities have incinerators |
| **Vaccine (service) delivery** |  |  |  |  |
| Proportion of vaccines delivered at health facilities per year | 40% | — | — | Costing workshop |
| Proportion of vaccines delivered at school-based events per year | 55% | — | — | Costing workshop; ring fencers data estimate 17 school-based events per year |
| Proportion of vaccines delivered at mobile events per year | 5% | — | — | Costing workshop; ring fencers data estimate 25 mobile events per year |
| Costs per minute of vaccinators' time to vaccinate one girl at a health facility | $0.05 | 15 min per vaccinee | — | Ring fencer data and government salary scale (TSH 54,096/day) |
| Cost per minute of vaccinators' time to vaccinate girls (regardless of number) at school-based event | $0.05 | 254 mins of vaccinator time | per event | Ring fencer data and government salary scale (TSH 54,096/day) |
| Cost of vaccinators' time to vaccinate girls (regardless of number) at a mobile event | $23.64 | 2.2 nurse days | per event | Ring fencer data and government salary scale (TSH 54,096/day); assumes 2 vaccinators for 1-day event, with 0.2 days allocated to travel time |
| Allowance per vaccinator for school-based vaccination (including travel) | $11.25 | 1.62 nurses | per event | Ring fencer data; allowance includes travel and extra duty allowance |
| Allowance per teacher to assist with vaccination | $5.68 | 1 teacher | per event | Ring fencer data |
| Allowance for vaccinator to travel to/from mobile event | $8.74 | 2 nurses | per event | Costing workshop; assumes 20,000 TSH roundtrip on a motorcycle |
| Per diem (extra duty) for vaccinator to attend mobile event | $17.48 | 2 nurses | per event | Costing workshop |
| Cotton pads (500 gm roll) | $1.40 | 1% for HPV | — | Costing workshop and UNICEF supply catalog |
| **Yearly Council Training** |  |  |  |  |
| Number of health councils in Tanzania | N/A | 184 health councils | — | Costing workshop; assumes 1 training per each of 184 health councils per year |
| Cost for health care provider to attend yearly 1-day council training | $23.64 | 30 providers | 60% | Costing workshop; assumes 30 health facilities per council, 1 attendee from each of 30 health facilities in attendance, 60% of day for HPV training; uses government salary scale (TSH 54,096/day) |
| Cost for Council Immunization Officer to attend yearly training | $52.44 | 1 officer | 60% | Costing workshop; assumes 60% of day for HPV training; uses government salary scale (TSH 120,000/day) |
| Cost for Council Medical Officer to attend yearly training | $80.85 | 1 officer | 60% | Costing workshop; assumes 60% of day for HPV training; government salary scale (TSH 185,000/day) |
| Cost for Council Reproductive & Child Health Coordinator to attend yearly training | $52.44 | 1 officer | 60% | Costing workshop; assumes 60% of day for HPV training; government salary scale (TSH 120,000/day) |
| Cost for National Officer to attend yearly training | $52.44 | 1 officer | 60% | Costing workshop; assumes 60% of day for HPV training; government salary scale (TSH 120,000/day) |
| Cost for Regional Officer to attend yearly training | $52.44 | 1 officer | 60% | Costing workshop; assumes 60% of day for HPV training; government salary scale (TSH 120,000/day) |
| Cost for teachers to attend yearly training | $24.29 | 114 teachers | 60% | DIVO interview and regional microplanning budget; based on BEST 2016 report, there are ~21,000 schools; assuming teachers are equally distributed across 184 health councils (21,000 schools / 184 health councils = 114 teachers per council) |
| Allowance for health care provider to attend yearly council training (full day) | $8.74 | 30 providers | 60% | Costing workshop; Full day per diem (TSH 20,000/day) |
| Allowance for Council Immunization Officer to attend yearly council training (half day) | $26.22 | 1 officer | 60% | Costing workshop: Half day per diem (TSH 60,000/half day) |
| Allowance for Council Medical Officer to attend yearly council training (half day) | $40.42 | 1 officer | 60% | Costing workshop: Half day per diem (TSH 92,500/half day) |
| Allowance for Council Reproductive & Child Health Coordinator to attend yearly council training (half day) | $26.22 | 1 officer | 60% | Costing workshop: Half day per diem (TSH 60,000/half day) |
| Allowance for National Officer to attend yearly council training | $52.44 | 1 officer * 2 days | 60% | Costing workshop; assumes full day + travel day; 60% of day for HPV training; government salary scale (TSH 120,000/day) |
| Allowance for Regional Officer to attend yearly council training | $52.44 | 1 officer * 2 days | 60% | Costing workshop; assumes full day + travel day; 60% of day for HPV training; government salary scale (TSH 120,000/day) |
| Allowance for teachers to attend yearly training | $8.74 | 114 teachers | 60% | DIVO interview and regional microplanning budget |
| Refreshments | $3.28 | 149 attendees | 60% | Regional microplanning budget; assumes 35 participants and 114 teachers |
| Venue | $87.40 | 1 venue | — | Regional microplanning budget |
| Projector for training | $43.70 | 1 day | — | Costing workshop; assumes 1-day use of projector for HPV; projector is part of conference package |
| Travel reimbursement for training participants | $4.37 | 149 attendees | 60% | Regional microplanning budget; assumes 35 participants and 114 teachers; 13 vaccines |
| Training printouts | $0.22 | 35 participants | (1/13) for HPV materials | Costing workshop; assumes 35 participants, packet contains information for 13 vaccines (1/13 for HPV) |
| Presentation development time | $52.44 | 8% | — | Costing workshop; assumes 8% of time spent on HPV |
| **Council Cascade Training** |  |  |  |  |
| Number of Council Cascade Trainings per year | 8 | trainings | — | Costing workshop; 1 Council Cascade Training per year, with 23 councils attending per session (184 councils / 8 trainings = 23 councils attending each training) |
| Cost for Council Immunization Officer (DIVO) to attend yearly training | $52.44 | 23 officers x 5 days | 20% | Costing workshop; 5-day training, with 1 day (20%) devoted to HPV activities; government salary scale (TSH 120,000/day) |
| Cost for Council Medical Officer to attend yearly training | $80.85 | 23 officers x 5 days | 20% | Costing workshop; 5-day training, with 1 day (20%) devoted to HPV activities; government salary scale (TSH 185,000/day) |
| Cost for Council Reproductive & Child Health Coordinator to attend yearly training | $52.44 | 23 officers x 5 days | 20% | Costing workshop; 5-day training, with 1 day (20%) devoted to HPV activities; government salary scale (TSH 120,000/day) |
| Cost for regional coordinators (RIVO, RMO, RRCHO) to attend yearly training | $52.44 | 3 officers | 1 day | Costing working; 1 full day training for HPV activities; government salary scale (TSH 120,000/day) |
| Allowance for Council Immunization Officer (DIVO) to attend yearly council training | $52.44 | 23 officers x 7 days | 20% | Costing workshop; 5-day training, plus 2 days of travel, 20% total allowance allocated to HPV activities; government salary scale (TSH 120,000/day) |
| Allowance for Council Medical Officer to attend yearly council training | $52.44 | 23 officers x 7 days | 20% | Costing workshop; 5-day training, plus 2 days of travel, 20% total allowance allocated to HPV activities; government salary scale (TSH 120,000/day) |
| Allowance for Council Reproductive & Child Health Coordinator to attend yearly council training | $52.44 | 23 officers x 7 days | 20% | Costing workshop; 5-day training, plus 2 days of travel, 20% total allowance allocated to HPV activities; government salary scale (TSH 120,000/day) |
| Allowance for regional coordinators (RIVO, RMO, RRCHO) to attend yearly training | $52.44 | 3 officers | 1 day | Costing working; 1-day allowance; government salary scale (TSH 120,000/day) |
| Conference package (including venue) | $30.59 | 80.6 participants per training | 20% | Costing workshop; includes venue rental; total participants = 184 * 3 participants per district/council + 31 regions * 3 regional officers; distributed across 8 trainings; 20% for HPV |
| **National-Level Supervision** |  |  |  |  |
| Number of supervision visits per year | 2 | visits | — | Costing workshop; goal is quarterly visits, but 2 visits/year are assumed based on current reported frequency |
| Costs for National Level Program Officer | $52.44 | 2 officers | 14 days * (1/7) | Costing workshop; multiplied by (1/7) to reflect the proportion of time spent on HPV-specific checklists; assumes 2 program officers per visit, with 2 visits per year; government salary scale (TSH 120,000/day) |
| Costs for Implementing Partner | $78.66 | 1.5 officers | 14 days * (1/7) | Costing workshop: CHAI, UN; Salary is unknown; multiplied by (1/7) to reflect the proportion of time spent on HPV-specific checklists; estimated to be 1.5x national level (TSH 180,000/day), with 1-2 officers |
| Costs for Regional Immunization Officer | $52.44 | 1 officer | 14 days * (1/7) | Costing workshop; 1-day visit; multiplied by (1/7) to reflect the proportion of time spent on HPV-specific checklists; government salary scale (TSH 120,000/day) |
| Costs for Regional Medical Officer | $52.44 | 0.5 officer | 14 days * (1/7) | Costing workshop; 1-day visit, but not always in attendance so 0.5x salary assumed; multiplied by (1/7) to reflect the proportion of time spent on HPV-specific checklists; government salary scale (TSH 185,000/day) |
| Costs for District Level Immunization Officer | $52.44 | 3 officers | 3 days *(1/ 7) | Costing workshop; 3 officers for 3 days; multiplied by (1/7) to reflect the proportion of time spent on HPV-specific checklists; government salary scale (TSH 120,000/day) |
| Costs for Health Care Provider | $39.33 | 1.5 officers | 4 health facilities * (1/7) | Costing workshop; 1-day visit; multiplied by (1/7) to reflect the proportion of time spent on HPV-specific checklists; government salary scale (TSH 90,000/day) |
| Costs for Driver | $19.67 | 1 driver | 7 days | Costing workshop; 1-day visit; government salary scale (TSH 45,000/day) |
| Costs for Administrative Staff | $39.33 | 1 staff | 7 days | Costing workshop; 7-day contribution of admin person; government salary scale (TSH 90,000/day) |
| Allowance for National Level Program Officer | $52.44 | 2 officers | 14 days * (1/7) | Costing workshop; multiplied by (1/7) to reflect the proportion of time spent on HPV-specific checklists; assumes 2 program officers per visit, with 2 visits per year; government salary scale (TSH 120,000/day) |
| Allowance for Implementing Partner | $78.66 | 1.5 officers | 14 days * (1/7) | Costing workshop: CHAI, UN; Salary is unknown; multiplied by (1/7) to reflect the proportion of time spent on HPV-specific checklists; estimated to be 1.5x national level (TSH 180,000/day), with 1-2 officers |
| Allowance for Regional Immunization Officer | $52.44 | 1 officer | 14 days * (1/7) | Costing workshop; 1-day visit; multiplied by (1/7) to reflect the proportion of time spent on HPV-specific checklists; government salary scale (TSH 120,000/day) |
| Allowance for Regional Medical Officer | $52.44 | 0.5 officer | 14 days * (1/7) | Costing workshop; 1-day visit, but not always in attendance so 0.5x salary assumed; multiplied by (1/7) to reflect the proportion of time spent on HPV-specific checklists; government salary scale (TSH 185,000/day) |
| Allowance for District Level Immunization Officer | $52.44 | 3 officers | 3 days * (1/7) | Costing workshop; 1-day visit; multiplied by (1/7) to reflect the proportion of time spent on HPV-specific checklists; government salary scale (TSH 120,000/day) |
| Allowance for Driver | $43.70 | 1 driver | 7 days | Costing workshop; 7 days; government salary scale (TSH 100,000/day) |
| Allowance for Administrative Staff | $39.33 | 1 staff | 7 days | Costing workshop; 7-day contribution of admin person; government salary scale (TSH 90,000/day) |
| Supplies: Supervision checklist | $0.04 | 148 pages | — | Costing workshop; 100 TSH per 148-page checklist |
| Supplies: Stationery | $4.37 | 0.9 | — | Costing workshop; 10,000 TSH lumpsum |
| Supplies: Tally sheets and patient vaccination cards | $87,657.26 | 0.25 | — | Costing workshop |
| Supplies: HPV registration sheet | $1.75 | 7151 sheets | — | Costing workshop; given to each health facility and school |
| Travel: flights | $699.24 | 7 regions | * (1/7) | Costing workshop; TSH 1,600,000/roundtrip flight to 7 regions for which flights are needed; divided by 7 to reflect proportion of supervision costs spent on HPV-specific activities |
| Travel: car fuel for inter-regional driving | $0.15 | 24 regions | 1,200 km * (1/7) | Distances from costing workshop and other car costs from MITU; assumes 1,200 km roundtrip; 24 regions (does not include 7 regions for which flights are needed); multiplied by (1/7) to reflect proportion of supervision costs spent on HPV-specific activities |
| Travel: car fuel for intra-district driving | $0.15 | 24 regions | 70 km * 14 days * (1/7) | Costing workshop; assumes 70km/day for 14 days multiplied by (1/7) to reflect proportion of supervision costs spent on HPV-specific activities |
| Travel: cost per day of car use | $57.52 | 14 days | * (1/7) | Distances from costing workshop and other car costs from MITU; supervision visits are 14 days multiplied by (1/7) to reflect proportion of supervision costs spent on HPV-specific activities |
| **Regional-Level Supervision** |  |  |  |  |
| Number of supervision visits per year | 2 | visits | — | Costing workshop; council supervision 2 visits/year, 3 days per district |
| Costs for Regional Immunization Officer | $52.44 | 1 officer | 3 days * (1/7) | Costing workshop; 3-day visit; multiplied by (1/7) to reflect the proportion of time spent on HPV-specific activities; government salary scale (TSH 120,000/day) |
| Costs for Regional Medical Officer | $80.85 | 1 officer | 3 days * (1/7) | Costing workshop; 3-day visit; multiplied by (1/7) to reflect the proportion of time spent on HPV-specific activities; government salary scale (TSH 185,000/day) |
| Costs for Council Level Immunization Officer | $52.44 | 3 officers | 3 days * (1/ 7) | Costing workshop; 3-day visit; multiplied by (1/7) to reflect the proportion of time spent on HPV-specific activities; government salary scale (TSH 120,000/day) |
| Costs for Health Care Provider | $39.33 | 4 providers | 3 days * (1/4) * (1/7) | Costing workshop; 3-day visit across 4 health facilities; multiplied by (1/7) to reflect the proportion of time spent on HPV-specific activities; government salary scale (TSH 90,000/day) |
| Costs for Driver | $19.67 | 1 driver | 3 days * (1/ 7) | Costing workshop; 3-day visit; government salary scale (TSH 45,000/day) |
| Allowance for Regional Immunization Officer | $52.44 | 1 officer | 3 days * (1/7) | Costing workshop; 3-day visit; multiplied by (1/7) to reflect the proportion of time spent on HPV-specific activities; government salary scale (TSH 120,000/day) |
| Allowance for Regional Medical Officer | $80.85 | 1 officer | 3 days * (1/7) | Costing workshop; 3-day visit; multiplied by (1/7) to reflect the proportion of time spent on HPV-specific activities; government salary scale (TSH 185,000/day) |
| Allowance for District Level Immunization Officer | $52.44 | 3 officers | 3 days * (1/ 7) | Costing workshop; 3-day visit; multiplied by (1/7) to reflect the proportion of time spent on HPV-specific activities; government salary scale (TSH 120,000/day) |
| Allowance for Driver | $43.70 | 1 driver | 3 days * (1/ 7) | Costing workshop; 3-day visit; multiplied by (1/7) to reflect the proportion of time spent on HPV-specific activities; government salary scale (TSH 100,000/day) |
| Supplies: Supervision checklist | $0.44 | 5 checklists | 3 facilities * (1/7) | Costing workshop; five 10-page checklists per health facility, 3 health facilities per council; 1,000 TSH per 10-page packet |
| Supplies: Stationery | $4.37 | 1 unit | * (1/7) | Costing workshop; 10,000 TSH lumpsum, multiplied by (1/7) to reflect the proportion of time spent on HPV-specific activities |
| Travel: car fuel for intra-regional driving | $0.15 | 3 days | 70 km * (1/7) | Costing workshop; assumes 70km/day for 3 days multiplied by (1/7) to reflect proportion of supervision costs spent on HPV-specific activities |
| Travel: cost per day of car use | $57.52 | 3 days | * (1/7) | Distances from costing workshop and other car costs from MITU; assumed 3-day visits; multiplied by (1/7) to reflect proportion of supervision costs spent on HPV-specific activities |
| **Cold chain and distribution** |  |  |  |  |
| **National level (n=1)** |  |  |  |  |
| Cold room (annualized cost) | $3,507.73 | 22% | — | Costing workshop; vaccines are stored in walk-in cold rooms at the Medical Stores Department at the national level; the Ministry of Health pays for space at MSD; HPV vaccines account for 22% of volume |
| **Regional level (n=31)** |  |  |  |  |
| Regional cold room (annualized cost) | $3,451.18 | 22% | — | Costing workshop; each region has 1 cold room; 22% allocated to HPV |
| Freezer tags | $7.72 | 22% | — | Costing workshop; pre-existing freezer tags for temperature tracking; 22% allocated to HPV |
| Refrigerator tags | $43.53 | 22% | — | Costing workshop; pre-existing refrigerator tags for temperature tracking; 22% allocated to HPV |
| Annual fuel costs | $1.09 | 5 liters/day | 365 days | Costing workshop; fuel (TSH 2,500/L) |
| Annual electricity costs (including solar) | $6,817.56 | 1% for HPV | — | Costing workshop; assumed to be 15,600,000/year, 1% allocated to HPV; monthly bill ranges from TSH 800k to 1.3m |
| Cold room maintenance | $175.39 | 22% | — | Costing workshop; estimated at 5% of annualized cold room costs; 22% allocated to HPV |
| Generators | $0.48 | 1% | — | Costing workshop and online search; assumes 1 generator per region (TSH 1,097.51/generator based on online search); 1% allocated to HPV |
| Costs for Vaccine distribution by Regional Immunization Vaccine Officer (RIVO) | $52.44 | 3 days x 4 times/year | 22% | Costing workshop; vaccine distribution, government salary scale (TSH 120,000/day); 22% allocated to HPV |
| Costs for Driver to accompany RIVO | $19.67 | 3 days x 4 times/year | 22% | Interview with RIVO; vaccine distribution, government salary scale (TSH 45,000/day); 22% allocated to HPV |
| Allowances for "extra duty" for RIVO | $52.44 | 3 days x 4 times/year | 22% | Interview with RIVO; government salary scale (TSH 120,000/day); 22% allocated to HPV |
| Per diem for Driver | $43.70 | 3 days x 4 times/year | 22% | Interview with RIVO; vaccine distribution, government salary scale (TSH 100,000/day); 22% allocated to HPV |
| **Council level (n=184)** |  |  |  |  |
| Refrigerator (annualized cost) | $485.01 | 22% | — | Costing workshop; 22% allocated to HPV |
| Freezer tags | $7.72 | 22% | — | Costing workshop; pre-existing freezer tags for temperature tracking; 22% allocated to HPV |
| Refrigerator tags | $43.53 | 22% | — | Costing workshop; pre-existing refrigerator tags for temperature tracking; 22% allocated to HPV |
| Annual fuel costs | $1.09 | 5 liters/day | 365 days | Costing workshop; fuel (TSH 2,500/L), 1% allocated to HPV |
| Annual electricity costs (including solar) | $6,817.56 | 1% for HPV | — | Costing workshop; assumed to be 15,600,000/year, 1% allocated to HPV; monthly bill ranges from TSH 800k to 1.3m |
| Cold room (annualized cost) | $24.25 | 22% | — | Costing workshop; estimated at 5% of annualized cold room costs; 22% allocated to HPV |
| Generators | $0.48 | 1% | — | Costing workshop; assumes 1 generator per region (TSH 1,097.51/generator based on online search); 1% allocated to HPV |
| Costs for Vaccine distribution by DIVO | $52.44 | 4 days x 12 times/year | 22% | Costing workshop; vaccine distribution, 22% allocated to HPV |
| Costs for Vaccine distribution by Assistant DIVO | $52.44 | 4 days x 12 times/year | 22% | Costing workshop; vaccine distribution, 22% allocated to HPV |
| Costs for Driver to accompany DIVO | $19.67 | 4 days x 12 times/year | 22% | Interview with RIVO; vaccine distribution, 22% allocated to HPV |
| Travel allowance: "extra duty" for DIVO | $19.67 | 12 times/year | 22% | Interview with RIVO; TSH 45,000 assumed per trip (reported range is range is TSH 30,000-65,000/trip); 22% allocated to HPV |
| Travel allowance: "extra duty" for Assistant DIVO | $19.67 | 12 times/year | 22% | Interview with RIVO; TSH 45,000 assumed per trip (reported range is range is TSH 30,000-65,000/trip); 22% allocated to HPV |
| Per diem for Driver | $43.70 | 12 times/year | 22% | Interview with RIVO; government salary scale (TSH 100,000/day); vaccine distribution, 22% allocated to HPV |
| **Health facility level (n=6,532)** |  |  |  |  |
| Refrigerator (annualized cost) | $408.19 | 22% | — | Costing workshop; 22% allocated to HPV |
| Incinerator (annualized cost) | $366.08 | 22% | — | Costing workshop; an estimated 2/3 of health facilities have an incinerator; 22% allocated to HPV |
| Freezer tags | $7.72 | 22% | — | Costing workshop; pre-existing freezer tags for temperature tracking; 22% allocated to HPV |
| Refrigerator tags | $43.53 | 22% | — | Costing workshop; pre-existing refrigerator tags for temperature tracking; 22% allocated to HPV |
| Gas cylinders | $0.05 | 50% | 1% for HPV | Costing workshop; pre-existing; estimated that 50% of health facilities use gas cylinders (115 TSH/cylinder), 1% allocated to HPV |
| Annual fuel costs | $1.09 | 5 liters/day | 365 days * 1% for HPV | Fuel (TSH 2,500/L), 1% allocated to HPV |
| Annual electricity costs (including solar) | $6,817.56 | 1% | — | Costing workshop; assumed to be 15,600,000/year, 1% allocated to HPV; monthly bill ranges from TSH 800k to 1.3m |
| Cold room (annualized cost) | $20.41 | 22% | — | Costing workshop; estimated at 5% of annualized cold room costs; 22% allocated to HPV |
| Generators | $0.48 | 1% | — | Costing workshop: Assumes 1 generator per region (TSH 1,097.51/generator based on online search); 1% allocated to HPV |
| Costs for Healthcare Provider | $23.64 | 1.5 officers | 12 trips x 30% for HPV | Cosing workshop; government salary scale (TSH 54,096/day); assumes 1.5 officers making 12 trips x 30% time for HPV |

**Supplemental Table 2.** C4P tool inputs for sensitivity analysis

|  | **Base case scenario**  **(source)** | **Best case scenario**  **(source)** | **Worst case scenario**  **(source)** |
| --- | --- | --- | --- |
| Vaccine coverage dose one^1^ | 2018: 59%  2019: 78%  2020: 82%  2021: 85%  2022: 87% | 2018: 59%  2019: 78%  2020: 85%  2021: 90%  2022: 95% | 2018: 59%  2019: 78%  2020: 78%  2021: 78%  2022: 78% |
| HPV1 to HPV2 drop-out rates^2^ | 2018: 34%  2019: 37%  2020: 30%  2021: 20%  2022: 10% | 2018: 34%  2019: 37%  2020: 12.2%  2021: 11.8%  2022: 8.0% | 2018: 34%  2019: 37%  2020: 37%  2021: 37%  2022: 37% |
| Service delivery – time per vaccination in health facility | 15 minutes (ring fencer observation) | 5 minutes (costing workshop assumption; rather group sessions) | 20 minutes (ring fencer observation; individual sessions) |
| Service delivery – time per vaccination in schools | 254 minutes (ring fencer observation; 1.62 persons) | 157 minutes (ring fencer observations; assumes 1 person) | 480 minutes (costing workshop, assumes 1 working day) |
| Average number of vaccinations per school vaccination session | 17  (Ring fencers data) | 24  Total number of girls per school* Proportion of girls vaccinated in schools / total number of schools | 10  (costing workshop) |
| Training of health care providers | First year only  (according to DIVO/RIVO) |  | Every year  (costing workshop) |
| Sensitization | First year only  (DIVO/RIVO) |  | Every year  (costing workshop) |
| Life-expectancy of refrigerators/cold-rooms | 15 | 20 | 10 |
| Annual discount rate | 0.03  (international standard) | 0.00  (international standard) | 0.07  (Bank of Tanzania 2019) |

^1^Actual numbers for 2018 and 2019: 59% and 78%; base case: for 2020–2022 based on costing workshop; best case: for 2020–2022 based on best achievements in other countries; worst case: for 2020–2022 assuming no improvement after 2019.

^2^Actual numbers for 2018 and 2019: 34% and 37% based on EPI DMT report (first program year=2018); base case: for 2020–2022 based on extrapolation of existing data to goals set by costing workshop; best case: for 2020–2022 based on costing workshop; worst case: for 2020–2022 assuming no improvement after 2019.
